# MicroRNA profiling of pancreatic ductal adenocarcinoma (PDAC) reveals signature expression related to lymph node metastasis

**DOI:** 10.18632/oncotarget.26804

**Published:** 2019-04-05

**Authors:** Moran Lemberger, Shelly Loewenstein, Nir Lubezky, Eran Nizri, Metsada Pasmanik-Chor, Eli Barazovsky, Joseph M. Klausner, Guy Lahat

**Affiliations:** ^1^ Laboratory of Surgical Oncology, Tel-Aviv Sourasky Medical Center, Tel-Aviv, Israel; ^2^ Division of Surgery, Tel-Aviv Sourasky Medical Center, Tel-Aviv, Israel; ^3^ Sackler Faculty of Medicine, Tel-Aviv University, Tel-Aviv, Israel; ^4^ Bioinformatics Unit, Tel Aviv University, Tel Aviv, Israel; ^5^ Institute of Pathology, Tel-Aviv Sourasky Medical Center, Tel-Aviv, Israel; ^6^ The Nikolas and Elizabeth Shlezak Cathedra for Experimental Surgery, Sackler Faculty of Medicine, Tel-Aviv University, Tel-Aviv, Israel

**Keywords:** microRNA, pancreatic cancer, lymph node metastasis, epithelial-to-mesenchymal transition (EMT), invasion

## Abstract

Lymph node (LN) metastasis occurs frequently in pancreatic ductal adenocarcinoma (PDAC), representing an advanced disease stage and independently predicting patient survival. Current nodal staging is inadequate preoperatively and even less so postoperatively, and molecular biomarkers are needed to improve prognostication and selection of therapy. Recent data have suggested important roles of miRNAs in PDAC tumorigenesis and progression. The aim of the present study was to identify miRNA expression signature for nodal spread in PDAC patients. Using PDAC human tissue specimens, we identified 39 miRNAs which were differently expressed in LN positive compared to LN negative PDAC samples. Of them, six miRNAs have been reported to play a role in cancer invasion and metastasis. A high versus low six- miRNA signature score was predictive of LN metastasis in the PDAC validation cohort. We demonstrated a similar expression pattern of four out of the six miRNAs in the plasma of PDAC patients. The results of our in-vitro studies revealed that miR-141 and miR-720 are involved in the process of epithelial to mesenchymal-transition in PDAC. These miRNAs significantly inhibited *in vitro* proliferation, migration and invasion of PDAC cells as evidence by gain- and loss- of function studies, specifically, via ZEB-1 and TWIST1 transcription factors, as well as through the activation of the MAP4K4/JNK signaling pathway.

## INTRODUCTION

Pancreatic ductal adenocarcinoma (PDAC) is the 3rd leading cause of cancer-related mortality in the United States and 8^th^ worldwide [1]. Despite radical surgery and adjuvant therapies, overall prognosis has not been changed over the last decades. Most of the tumors recur, distant metastasis is not uncommon, and 5-year survival of completely resected patients is only up to 25% [2]. Of all the clinical and pathological factors that are associated with adverse prognosis, lymph node (LN) involvement is the most important, especially following curative surgery [3]. The current staging of LN metastasis depends on the extent of lymphadenectomy performed by the surgeon and the adequacy of the pathological evaluation. Since both are operator-dependent, the accuracy of postoperative nodal staging is suboptimal. Moreover, preoperative staging for nodal involvement is based solely on imaging studies which is far from precise for the purpose. Thus, there is a clear need for an objective and a more accurate means for the appraisal of nodal status. To date, various molecules are used in cancer diagnosis, prediction of outcome, and therapy. They include mismatch repair genetic mutations in hereditary colorectal cancer [4], Ki67 in neuroendocrine tumors [5], and c-kit mutations in gastrointestinal stromal tumors [6].

We had shown that micro RNAs (miRNAs) play a role in the progression of intraductal papillary mucinous neoplasm (IPMN) of the pancreas [7]. MiRNAs are small, noncoding RNAs that bind messenger RNAs of hundreds of genes, resulting in degradation of the target messenger RNA or inhibition of translation [8]. Aberrant expression of miRNAs contributes to carcinogenesis by promoting the expression of proto-oncogenes or by inhibiting the expression of tumor suppressor genes in PDAC [9]. While the role of miRNAs in tumor growth, apoptosis, invasion and migration is well documented, little is known about their potential role in lymphatic spread.

Our aim was to identify specific miRNAs in the tumors and plasma of primary PDAC which are associated with the presence of LN metastasis in patients who had undergone curative surgery at our institution.

## RESULTS

### Differential expression of human miRNA in LN positive (N1) compared to LN negative (N0) PDAC and normal pancreatic tissues

**Figure 1:**
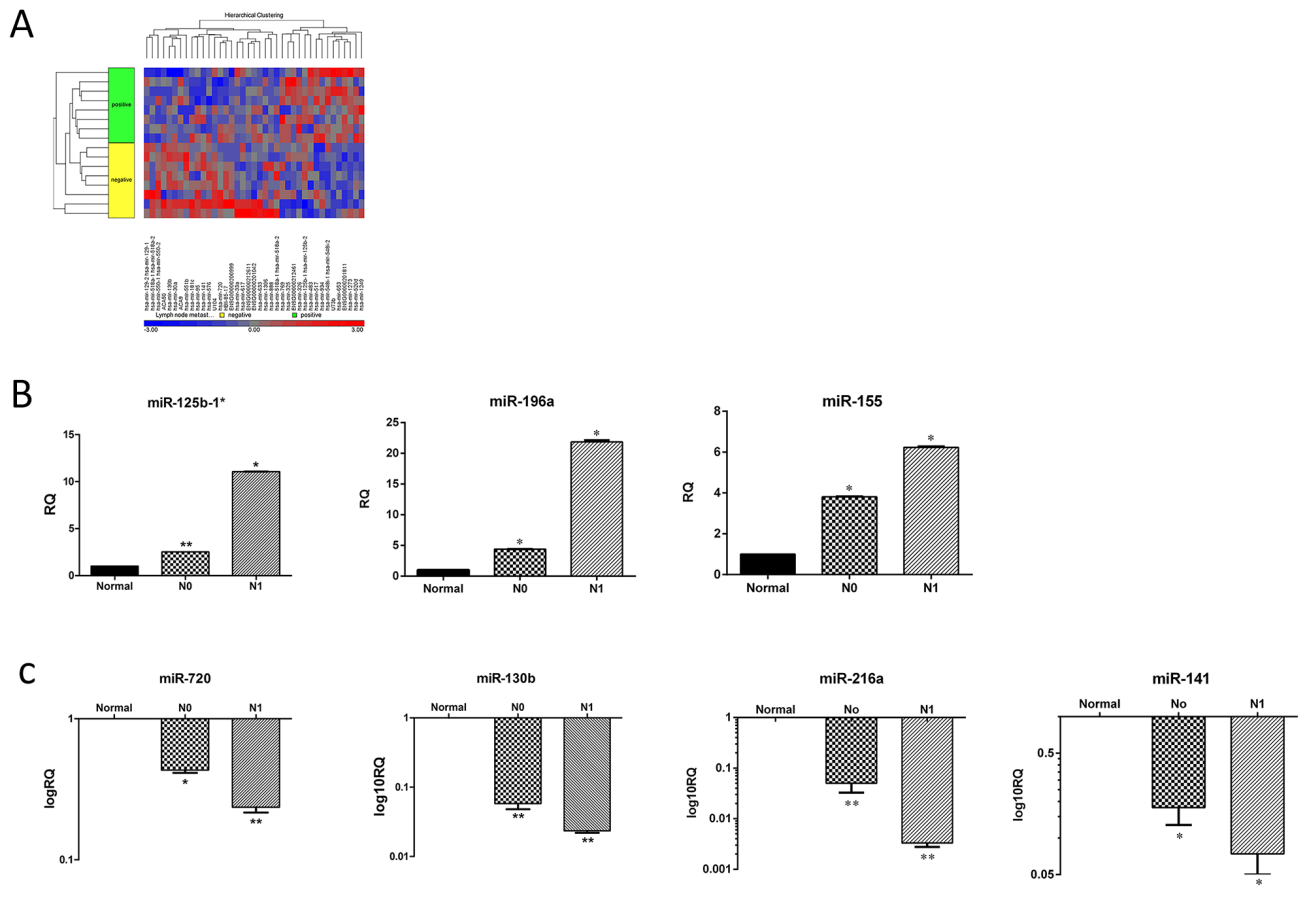
miRNA expression pattern in N1 and N0 PDAC samples **(A)** Affymetrix microarray hierarchical clustering performed on miRNA of LN positive (N1) compared to LN negative (N0) PDAC samples (p<0.05). A colored bar indicating the standardized log2 intensities accompanies the expression profile. **(B)** qRT-PCR validation of the expression levels of three representative up regulated miRNA: hsa-miR-125-b*, hsa-miR-196a, hsa-miR-155. **(C)** qRT-PCR validation of the expression levels of four representative down regulated miRNA: hsa-miR-720, hsa-miR-130b, hsa-miR-216a and hsa-miR-141 from the indicated groups: Normal, N0 and N1. Each histogram represents the average of 8 samples as indicated in materials and methods (n=8). ^*^P <0.05; ^**^P < 0.01; ^***^P <.0001.

First, we chose 24 pancreatic specimens (16 PDAC and 8 normal pancreas tissues) from our clinically annotated tissue bank to assemble a representative cohort of surgically treated PDAC patients; Table 1 summarizes their clinical and pathological characteristics. Of this cohort, 8 PDAC patients had nodal involvement (N1) and 8 had not (N0). All samples included in the present study were suitable for the miRNA analysis. Hierarchical clustering was carried out to detect potential clusters in the miRNA expression matrix (Figure 1A). Based on a cutoff value of fold change ≥2 and a false detection rate (FDR) <0.05, 39 miRNAs were significantly differentially expressed by N1 PDAC compared with N0 PDAC and normal pancreatic tissues (p< 0.05). Of them, 15 miRNAs were up-regulated and 24 were down-regulated in the cohort of N1 specimens. We then selected miR-141, miR-216a, miR-130b, miR-720, miR-155, miR-196a and miR-125b-1 for further validation studies, because of their particular role in pancreatic cancer [10–15]. Table 2 summarizes the log2-fold change of those miRNAs in the N1 PDAC samples compared to the N0 PDAC samples. In order to validate these data, we then performed qRT- PCR utilizing 24 new samples, 16 PDAC specimens and 8 normal pancreatic controls. The results of the qRT- PCR were in accordance with the microarray data (Figure 1B and 1C). The expression levels of each miRNA in the sub-cohorts of the N1 and N0 PDAC samples were compared with the expression levels of normal pancreatic tissues: miR-125b-1* (N1 = 11.059 ± 0.016 vs. N0 = 2.519 ± 0.002), miR-196a (N1 = 21.849 ± 0.369 vs. N0 = 4.383 ± 0.057), and miR-155 (N1 = 6.230 ± 0.020 vs. N0 = 3.089 ± 0.040) were up-regulated (Figure 1B), whereas miR- 720 (N1 = 0.236 ± 0.018 vs. N0 = 0.433 ± 0.038), miR-130b (N1 = 0.027±0.003 vs. N0 = 0.058±0.001), miR-216a (N1 = 0.003 ± 0.001 vs. N0 = 0.050 ± 0.002), and miR-141 (N1 = 0.074 ± 0.012 vs. N0 = 0.178 ± 0.018) were down-regulated (Figure 1C).

**Table 1:** Clinical and pathological patient characteristics

Characteristics	MiRNA array cohort (n=16)	Validation cohort (n=24)	MiRNA signature testing cohort (n=54)	Plasma miRNA cohort (n=20)^*^
**Gender**				
Male	6	14	28	12
Female	10	10	26	8
**Age, median (range)**	62 (44-84) years	64.5 (44-77) years	66 (38-82) years	64 (45-83) years
**Number of evaluated LN**				
Median (range)	12 (4-23)	20 (18-25)	16 (7- 28)	12 (1-24)
**Number of affected LN**				
0	8	13	19	10
1-3	6	2	22	8
4-9	2	9	13	2
**Differentiation**				
Well	1	0	6	6
Moderate	10	12	11	9
moderate to poor	0	5	9	2
Poor	4	7	22	0
Unknown	1	0	6	3
**Lymphovascular invasion**				
Yes	4	6	25	4
No	8	6	16	11
n/a	4	12	13	5
**Perineural invasion**				
Yes	9	17	40	14
No	2	3	5	4
n/a	5	4	9	2
**Tumor size, median (range)**	3 (1.2-8.5) cm	3 (2.1-6) cm	3.6 (0.4-5.8)	3 (1.5-4.5) cm

^*^ The plasma miRNA cohort includes 20 PDAC patients and 10 normal controls; clinical and pathological characteristics of this cohort represent the sub group of PDAC patients.

**Table 2:** MiRNAs with significantly different expression patterns between LN positive (N1) and LN negative (N0) pancreatic cancer tissues (P < 0.05)

MiRNA	Fold change (N1 vs. N0)
hsa- miR-125b-1^*^	3.191
hsa- miR-196a	2.585
hsa- miR-155	1.596
hsa- miR-720	-2.268
hsa- miR-130b	-1.743
hsa- miR-216a	-12.631
hsa- miR-141	-1.651

These data suggest that primary PDAC tumors of N1 vs. N0 patients exhibit different miRNA expression patterns. Next, we sought to evaluate whether these distinctive signatures might be used to predict the presence of PDAC lymphatic spread in a different cohort of surgically treated PDAC patients.

### A miRNA signature based on six miRNAs predicts the nodal status of PDAC patients

The expression of this specific subset of seven miRNAs was then re-validated in a different testing cohort of 54 PDAC samples which comprised 35 N1 patients and 19 N0 patients. Out of the seven miRNAs analyzed, only six (miR-196a, miR-155, miR-720, miR-130b, miR-216a, and miR-141) retained their discriminative significance between N1 and N0 specimens (P < 0.05). This six-miRNAs signature was then used to calculate a risk score for each patient. Patients were re ranked according to the number of aberrantly expressed miRNAs (Table 3), and then divided into high-risk (risk score >4) or low-risk (risk score ≤4) groups. A risk score was assigned for each patient according to a linear combination of the expression level of the miRNA weighted by the regression coefficient from the training samples. A risk score >4 was associated with an increased prevalence of nodal metastasis, 85.7% versus 36.8% in the cohort of patients with a risk score ≤4 (p=0.002; Table 4). The positive and negative predictive values of this miRNA expression signature model were 83.7% and 70.5%, respectively. These results suggest that this miRNA expression signature model can serve as a potential molecular predictive model of lymphatic metastasis in PDAC patients. We concluded that these data should be further validated prior to the possible implementation of the model in clinical practice. In order to facilitate its potential usage as a noninvasive clinical predictive tool, we next evaluated whether these miRNAs could be detected in the plasma of N1 vs. N0 PDAC patients.

**Table 3:** LN metastasis signature score was calculated as the number of aberrantly expressed miRNAs ranging from 0-6. A total score of 5/6 was considered as high risk for LN metastasis

miRNA	Points
miR-141	0/1
miR-155	0/1
miR-720	0/1
miR-216a	0/1
miR-196a	0/1
miR-130b	0/1

**Table 4:** LN metastasis signature score > 4 was more common in N1 vs. N0 patients (p=0.002) among the validation cohort of PDAC patients (n=54)

			LN metastasis		
			0	1	Total
LN metastasis signature	0	Count	12	7	19
Score>4		% within LN metastasis signature score>4	63.2%	36.8%	100%
	1	Count	5	30	36
		% within LN metastasis signature score>4	14.3%	85.7%	100%
	Total	Count	17	37	54
		% within LN metastasis signature score>4	31.5%	68.5%	100%

### Deregulations of circulating miRNAs in the plasma of N1 versus N0 patients correlate with their primary tumor expression patterns

**Figure 2:**
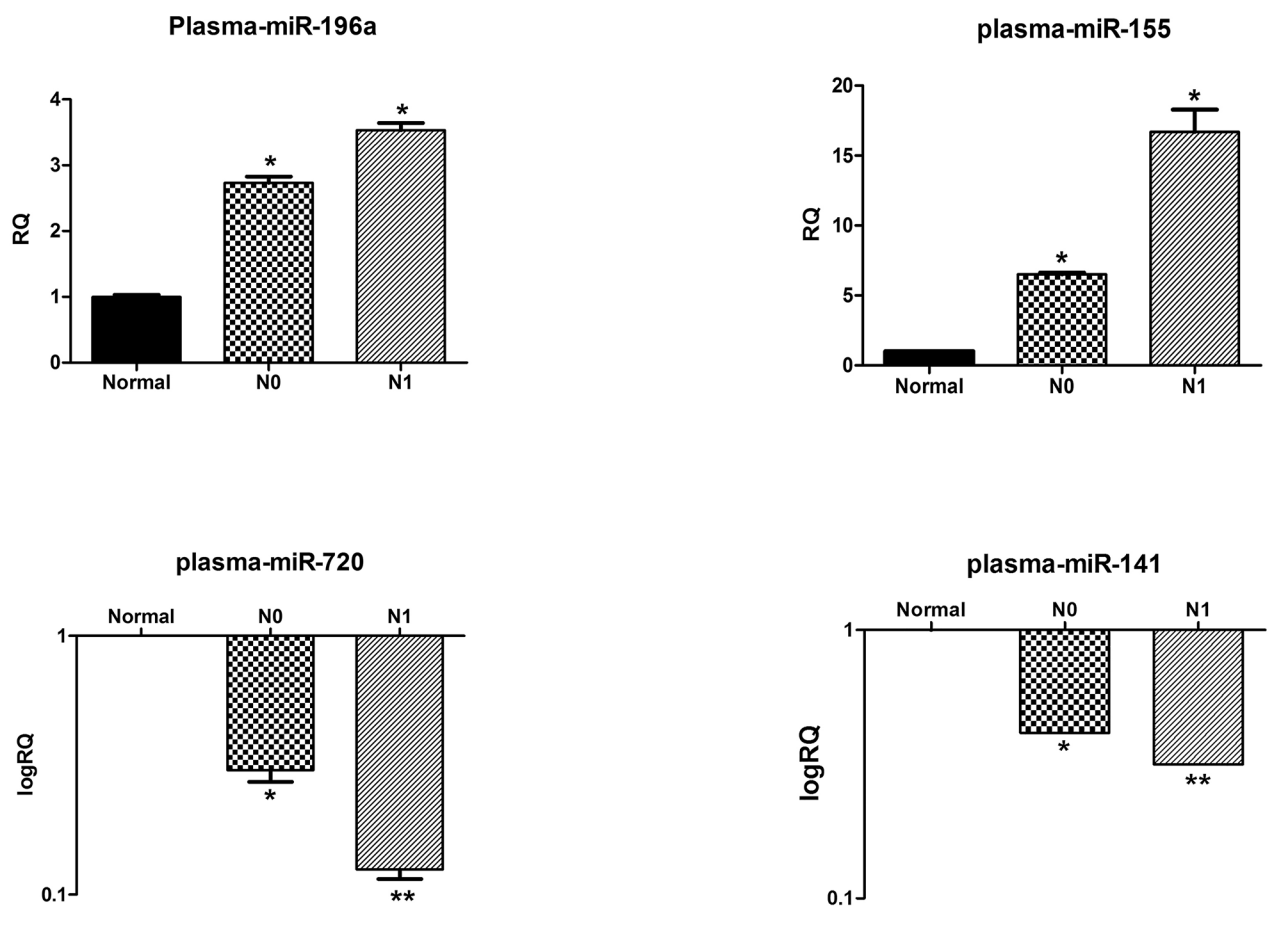
Plasma levels of selected miRNAs correlate with their primary tumor expression patterns The relative levels of miR-196a, miR-155 and miR-720 and miR-141 in the plasma of healthy controls, N1 and N0 pancreatic cancer patients were normalized to spike-in control cel-miR-39. Each histogram represents the average of 10 samples as indicated in materials and methods (n=10). ^*^P <0.05; ^**^P < 0.01; ^***^P <.0001.

Thirty plasma samples from N0 (n=10), and N1 (n=10) PDAC patients, as well as from healthy individuals (n=10) were collected and processed, and their levels of circulating miRNAs were analyzed by qRT-PCR. The miR-196a levels in the N1 and N0 PDAC patients were 3.513± 0.438-fold vs. 2.733 ± 0.294- fold, respectively (P < 0.05) (Figure 2). The MiR-155 levels were also significantly higher in the sub-cohort of N1 patients, 17.996 ± 0.757- fold vs. 6.504 ± 0.290-fold in the plasma of N0 patients (P < 0.05). In contrast, both miR-720 and miR-141 levels were significantly lower in the plasma of the N1 PDAC patients than in the plasma levels of N0 patients (miR-720 = 0.125 ± 0.026- fold vs. 0.303 ± 0.107- fold, respectively, P < 0.01 and miR-141 = 0.316 ± 0.0001- fold vs. 0.415 ± 0.0006-fold, respectively, P < 0.01). MiR-130b and miR-216a were dropped from the analysis since we were unable to amplify them by qRT-PCR with high specificity.

The correlation between the results obtained from the plasma and the miRNA expression patterns that we identified in the primary PDAC tumors strongly warranted further investigation of the exact role of miRNAs in the biology of pancreatic cancer nodal and systemic metastasis. Therefore, we next evaluated the *in vitro* effects of selected miRNAs on PDAC cells.

### MiR-141 and miR-720 up- regulation inhibits PDAC cellular growth, invasiveness, and chemoresistance

Both miR-141 and miR-720 were downregulated in N1 PDAC patients and were included in the high-risk expression signature. MiR-141 is well reported as being commonly dysregulated in malignant tumors and known to play essential roles in PDAC tumor development and progression [11, 16]. In contrast, miR-720 is scarcely studied and its role in pancreatic cancer is unknown.

**Figure 3:**
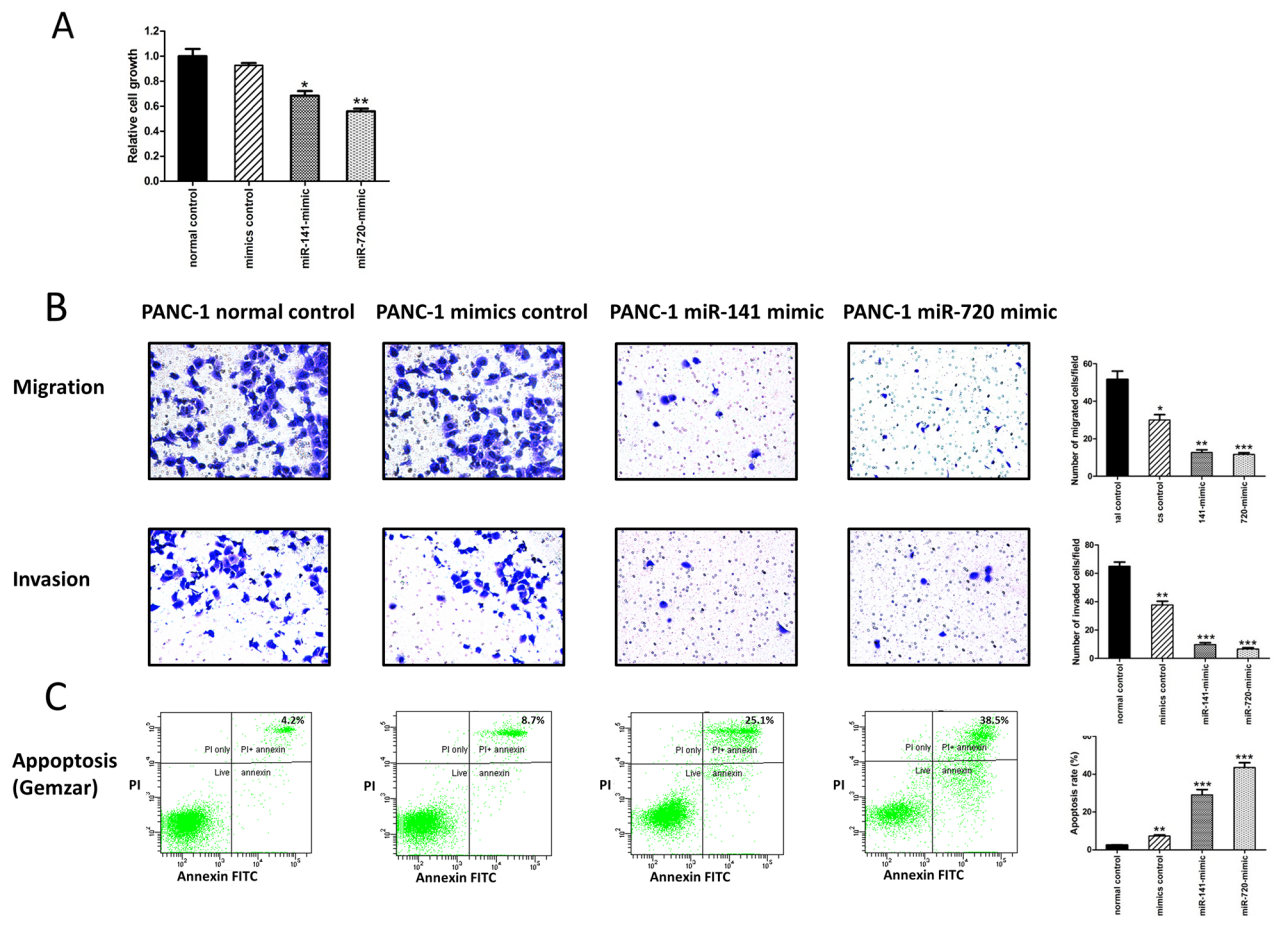
MiR-141 and miR-720 inhibits the tumorigenesis of PANC-1 cells **(A)** Assessment of cell proliferation by XTT assay. The proliferation of PANC-1 cells was significantly inhibited by overexpression of miR-141 and miR-720. **(B)** Overexpression of miR-141 and miR-720 markedly decreased migration and invasion of PANC-1 cells. Representative images are shown (magnification, X100), graphs represent the average of 3 repeated experiments ±SD. **(C)** Annexin-V/PI FACS analysis demonstrating a marked increase in the percentage of gemcitabine-induced apoptotic PANC-1cells following overexpression of miR-141 and miR-720. ^*^P <0.05; ^**^P < 0.01.

To explore the potential role of these miRNAs in PDAC tumor aggressiveness, we assessed their effects on cellular proliferation, motility, invasiveness, and resistance to conventional anti- PDAC chemotherapy. PANC-1 pancreatic cancer cells were initially transfected with a negative control, miR-141, as well as miR-720 mimics. The transfection of PANC-1 cells with miR-141 and miR-720 mimics significantly decreased their proliferative capability (Figure 3A) and reduced PANC-1 cellular migration and invasion (Figure 3B). We then evaluated the effect of these miRNAs on resistance to gemcitabine, a commonly used chemotherapeutic agent in pancreatic cancer patients. Following treatment of PANC-1 cells with increasing doses of gemcitabine (0.5-10 μM), cell viability was markedly reduced in the miR-141 and miR-720 mimic-transfected cells compared with the negative control group (Figure 3C depicts the 1 μM dose). These data demonstrate that up- regulation of both miRNAs results in reduced PDAC cellular aggressiveness, and increased chemosensitivity.

**Figure 4:**
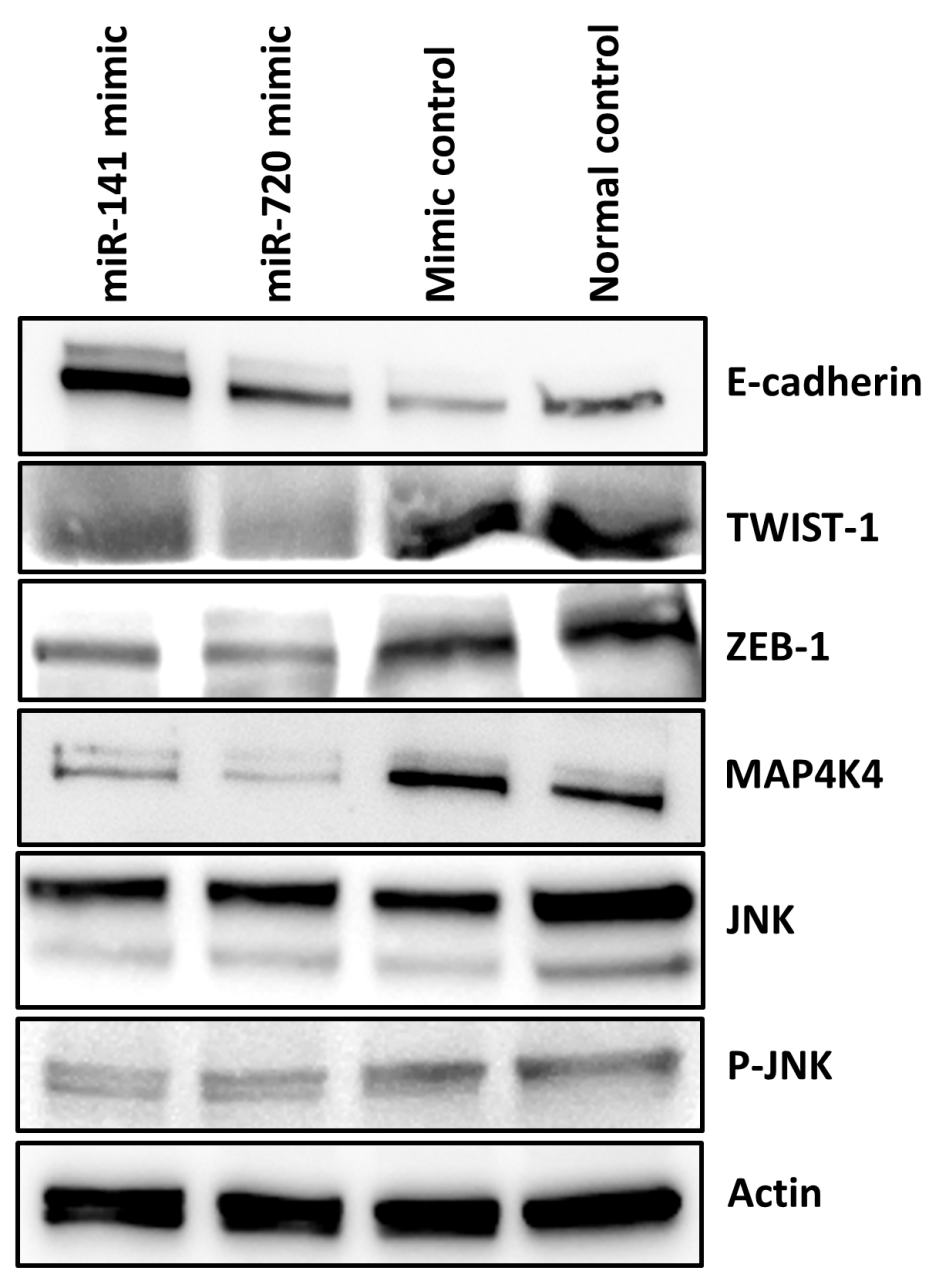
MiR-141 and miR-720 regulates EMT Western blot analysis indicated that enhanced expression of miR-141 and miR-720 is associated with decreased expression of EMT associated transcription factors ZEB-1and TWIST-1. As a result the expression level of E-cadherin was increased. Overexpression of miR-141 and miR-720 decreased the expression of MAP4K4 and the phosphorylation of JNK in PANC1 cells.

MiR-141 and miR-720, to a lesser extent, have been reported in relation to epithelial to mesenchymal transition (EMT) [17, 18]. Since EMT enables epithelial cells to acquire the abilities to invade, to resist chemotherapy, and to disseminate into distant organs, we next evaluated potential *in vitro* effects of these miRNAs on EMT characteristics in PDAC [19]; we studied the expression of EMT key regulators such as the ZEB and TWIST transcription factors, as well as the Ser/Thr kinase MAP4K4- c-Jun NH2- terminal kinase (JNK) protein kinase signaling pathway [11, 20, 21]. Overexpression of miR-141 and miR-720 suppressed the expression of both ZEB-1 and TWIST-1 resulting in up-regulation of E-cadherin, which was more prominent in the miR-141 transfected cells (Figure 4). Additionally, as depicted in Figure 4, overexpression of both miRNAs significantly decreased MAP4K4 expression resulting in decreased phosphorylation of JNK. These results imply that down- regulation of miR-141 and miR-720 may promote an aggressive phenotypic behavior of PDAC cells via EMT-associated pathways.

## DISCUSSION

MiRNAs are important regulators of various physiological and pathological processes including cancer, spurring extensive research on their potential application as biomarkers for cancer diagnosis, progression, and therapy [22]. In the present study, we investigated a potential association between miRNA expression and nodal metastasis in patients with pancreatic cancer. Microarray analysis identified several dysregulated miRNAs in those with N1 compared with N0 nodal status. Of them, seven miRNAs have been previously studied in cancer and, to some extent, specifically in pancreatic cancer [10, 11, 13, 23].

Our results suggest a signature of six miRNAs that is associated with lymphatic spread of PDAC; an expression of five or six of these molecules by the primary tumor is highly predictive for nodal metastases.

MiRNA expression signatures may be used to deliver a specific diagnosis. For instance, a signature of nine miRNAs was able to discriminate between normal and malignant breast tissues [24]. Similarly, Lu et al reported a signature of five miRNAs capable of providing a high diagnostic accuracy of hepatocellular carcinoma [25]. MiRNAs can also predict cancer progression and prognosis: a panel of 20 miRNAs was associated with an advanced disease stage and decreased overall survival of lung cancer patients [26]. Research on potential biomarkers for nodal metastasis is intensive and is being conducted on various types of cancers [27–29]. Two recent publications introduced a nomogram based on primary tumor miRNAs for lymph node metastasis in early breast cancer [30], and a signature expression for nodal involvement in T1 colorectal carcinoma [31]. In contrast, while numerous studies evaluated the prognostic value of different miRNAs in pancreatic cancer, little is known about their role as predictors of nodal spread [32].

To date, the preoperative diagnosis of pancreatic cancer nodal and distant metastasis is mostly based on imaging studies. However, despite enormous technical progress, their efficacy is still limited, and there is a pressing need for novel diagnostic tools. The detection of molecular indicators for pancreatic cancer progression is of great relevance in clinical practice. Specific molecular signatures for nodal, hematogenic, and intraperitoneal spread may enable clinicians to better select treatment strategies for their patients and improve the ability to predict their outcomes. Thus, PDAC patients with nodal spread may benefit from a more aggressive therapeutic approach, such as preoperative systemic therapy, while molecular markers suggesting remote metastasis can avoid unnecessary operations.

A substantial number of miRNAs are present in blood and other body fluids (“circulating microRNAs” -c-miRNAs). As biomarkers, c-miRNAs have considerable stability and resistance to endogenous RNase activity as well as to severe physicochemical conditions in body fluids, thereby making them attractive for use in clinical applications [33]. Moreover, c-miRNAs are easy to obtain without severe damage to the patient.

Our data indicate that four of the PDAC N1-associated miRNAs are circulating and could be detected in the plasma of N1- PDAC patients. Several studies evaluated different c-miRNA signatures as breast cancer molecular prognosticators [34], and predictors of disease recurrence and survival [35, 36]. Additionally, based on the observation that miR-10b and miR-373 are overexpressed in breast cancer lymph node metastases, circulating levels of these miRNAs were assessed in the sera for their potential utilization as biomarkers for detecting breast cancer lymph node metastases [37]. The authors reported higher levels of miR-10b and miR-373 in plasma from preoperative breast cancer patients with lymph node metastasis compared with patients without metastasis and normal controls [37]. Growing evidence also supports the use of c-miRNAs as diagnostic and prognostic biomarkers in pancreatic cancer [38]. Nevertheless, to the best of our knowledge, we are the first to describe an association between circulating miR-155, miR-141, miR-720, and miR-196a with lymph node metastasis in pancreatic cancer.

Encouraged by the reproducibility of our findings, we sought to further evaluate the biological relevance of these miRNAs in the context of metastatic spread. MiRNAs have emerged as integral components of almost every cancer-related biological process, including cellular differentiation, proliferation, migration, apoptosis, EMT, and angiogenesis [39]. EMT is a critical early step in the formation of tumor metastasis by controlling the detachment of invasive cancer cells from the primary tumor [40]. During that process, the cells become more invasive, thereby starting the multistep process of metastasis, or escape from apoptosis and senescence as well as immunosuppression [41]. There has been extensive investigation on the function of the miR-200 family in EMT, and miR-141, a member of this family of miRNAs, has a significant role in that process. Our *in vitro* data confirmed previous studies which demonstrated its role in the EMT of pancreatic cancer cells as well as its cellular downstream effects by targeting ZEB1 and TWIST1 [11, 42, 43]. Expression of miR-200 family members correlates positively with E-cadherin expression and negatively with the miR-200 target ZEB1. Interestingly, ZEB1 can also directly suppress the transcription of miR-200 family members, miR-141 and miR-200c [42], indicating a significant interplay between ZEB1 and miR-200 family miRNAs that contributes to the differentiation state of pancreatic cancer cells. Recent reports suggested that the miR- 200 family and, specifically, miR-141 may have a role in the lymphatic spread of various epithelial tumors, including breast, colorectal, thyroid, and gastric cancers [44–47]. An expression miRNA profile of human metastatic cancers, which includes miR-141, has also been proposed [48]. Taken together, it is reasonable to conclude that miR-141 is implicated in the acquisition of invasive and metastatic properties by PDAC cells, thus promoting the process of lymph node metastasis in pancreatic cancer. Moreover, because of its dysregulated level in the plasma of patients with PDAC and its key role in EMT, miR-141 should also be studied in the context of distant metastasis. Unlike miR-141, little is known about the role of miR-720 in EMT, lymphatic spread or PDAC biology. A recent report showed that miR-720 promotes *in vitro* cellular migration in cervical cancer [49]. Overexpression of miR-720 resulted in a decrease in E-cadherin and an increase in the expression of vimentin [49]. Similarly, deregulation of miR-720 was reportedly associated with EMT and metastasis in renal cell carcinoma [50]. In accord with these data, our results revealed that the deregulation of miR-720 expression also affects EMT in PDAC cells. However, in opposition to existing data, overexpression of miR-720 was associated with decreased levels of TWIST1 and ZEB1 and increased expressions of E-cadherin, as well as impaired proliferation, migration, and invasion. Clearly, there is inconsistency between our results and the scarce data on the role of miR-720 in cancer progression or EMT. However, our *in vitro* data match and support the clinical results of the array, thus, strengthening the paradigm that down-regulation of miR-720 results in enhanced EMT, and therefore in increased incidence of lymph node metastasis in PDAC. These data is in accord with the findings of Lin- Zi et al who showed that miR-720 inhibits tumor invasion and migration in breast cancer by targeting TWIST1 [17].

Finally, we explored that activation of the MAPK/JNK pathway by deregulation of miR-141 and miR-720 may explain, at least in part, the observed EMT features. MAP4K4 was previously described as a direct target of miR-141 [11] and as a promoter of EMT [51]. We show that up regulation of miR-141 and miR-720 result in decreased levels of MAP4K4 and reduced phosphorylation of JNK as well as EMT suppression in PDAC cells. While several reports have shown that MAP4K4 and JNK are related to EMT in various cancers [51, 52] there is no data concerning the whole axis including both miRNAs.

In summary, the presented data provide new insights into the process of EMT and pancreatic cancer lymphatic spread. We identified a miRNA expression signature that can discriminate between patients with PDAC who have nodal metastasis and those who do not, confirming a direct involvement of miRNAs in pancreatic cancer progression and specifically in lymph node metastasis. Moreover, the identification of several N1 signature miRNAs in the plasma of patients with PDAC may facilitate and establish their usage as a diagnostic tool. Searching for a potential mechanism, we investigated the role of select signature miRNAs in EMT, a critical step in cancer metastasis. We suggest that downregulation of miR-141 and miR-720 activates ZEB-1 and TWIST1 transcription factors as well as the MAPK/JNK signaling pathway. Taken together with our clinical observations and existing data, we propose a potential molecular mechanism that should be further studied in the context of pancreatic cancer lymphatic spread.

## MATERIALS AND METHODS

### Cell lines and patient tissue samples

PANC-1 human pancreatic adenocarcinoma cells were purchased from the American Tissue Culture Collection (ATCC). Cells were detected as *Mycoplasma*-free by PCR-based method (Hymicoplasma Detection Kit) and were cultured for no more than 20 passages between thawing and use in experiments. PANC-1 cells were cultured in Dulbecco’s modified Eagle’s medium (DMEM) supplemented with 10% fetal calf serum (FCS) and 100 U/ml penicillin-streptomycin (Biological Industries, Beit Haemek, Israel). The cells were maintained in a humidified 5% CO2 atmosphere at 37°C. All tumor blocks were from surgically resected primary pancreatic cancer with curative intent. We reviewed the medical records of all patients in our departmental database who were diagnosed as having PDAC. They were divided into four cohorts: one consisted of 16 PDAC patients who were evaluated for miRNA expression by comparing those who were node positive (N1) to those who were node negative (N0), 24 PDAC patients who served to validate the results obtained from the miRNA array, 54 PDAC patients who served to evaluate the predictive value of the suggested signature obtained in our initial analysis, and 30 PDAC plasma specimens collected before surgery. Adjacent normal pancreatic tissues from patients with PDAC served as the control tissue. None of the patients received neoadjuvant therapy. One part of the sample of each tumor specimen collected during surgery was always embedded in paraffin and the other was immediately snap-frozen and stored at -80°C until RNA extraction. A pathologist experienced in pancreatic pathology (E.B.) reviewed all of the histologic slides. All samples were collected with the informed consent of the patients, and the study was approved by the Human Ethics Review Committee of the Israeli Ministry of Health and the Tel-Aviv Sourasky Medical Center.

### MiRNA microarray

MiRNA microarray was conducted and analysed as described previously [7]. Briefly, formalin-fixed paraffin-embedded (FFPE) tumor samples were examined and the presence of IPMN and PDAC were confirmed. Ten μm-thick sections were cut and transferred onto glass slides. H&E-stained tissue was marked for margins of invasive and non-invasive tumor. Microdissection was performed manually. Tumors within the marked margins were removed and total RNA including small RNAs were extracted and measured (NanoDrop Technologies, Wilmington, DE) from each tissue by means of the miRNeasy FFPE kit (Qiagen) according to the manufacturer’s protocol. The miRNA microarray experiments were utilized for miRNA hybridization with the GeneChip^®^ miRNA Array (Affymetrix).

### Microarray analysis

MiRNA profiles were extracted from Affymetrix CEL files using Partek Genomics Suite (GS 6.5, Copyright 2010 [available: http://www.partek.com]; Partek Inc., St Louis, MO). Data were normalized and summarized with the Robust Multichip Average algorithm [53] and converted to log2 values. Batch removal was applied for the various processing dates. The resulting data were used for statistical analysis. One-factor analysis of variance (ANOVA) was performed to test for significant differences between the means of the analyzed groups. MiRNA expression data were sorted using cutoffs of P <.05 under false discovery rate correction for multiple comparisons [54] with an adjustment criteria and fold difference of 2. The panel of the most significantly differentially expressed miRNAs (fold-change >2 or ≤2 and P <.05) were determined and selected for further validation. Hierarchical cluster analyses were performed with Pearson correlation using the Partek Genomics Suite. MiRNA arrays were normalized, and the data were uploaded to http://www.ncrna.org/KnowledgeBase/link-database/mirna_target_database.

### Plasma sample collection

Whole blood samples were collected from patients with PDAC and healthy individuals (n=30) before surgery in EDTA-containing tubes (BD Vacutainer; Becton Dickinson and Company, Franklin Lakes, NJ) and subjected to centrifugation at 2000 x g for 10 minutes at room temperature. The upper plasma layer was immediately pooled after centrifugation, aliquoted into RNase-free tubes and stored at −70°C until use.

### Extraction of circulating miRNAs from plasma

Plasma miRNA was extracted from plasma (200 μl) using the miRNeasy mini kit (Qiagen GmbH, Hilden, Germany). A synthetic *C. elegans* miRNAs *cel*-miR-39 oligonucleotide RNA (25 fmol) (5′-UCACCGGGUGUAAAUCAGCUUG-3′) (Sigma-Aldrich) was added to all the plasma aliquots as an exogenous miRNA spiked-in control to allow for normalization of sample-to-sample variation in the RNA isolation procedure efficiency and all subsequent procedural steps, including qRT-PCR amplification [33].

### Quantitative reverse transcription real-time PCR

MicroRNA array results were validated via qRT-PCR. Quantitative analyses of miRNA levels were performed using the stem-loop TaqMan^®^ MicroRNA Assays kit (Applied Biosystems, Foster City, CA) as described previously [7, 55]. The non-coding small nuclear RNA U6 (U61973; Applied Biosystems) was used as internal control. Gene expression levels were quantified using the ABI StepOne Software v2.3, and relative fold expression was calculated using the comparative CT (2^−ΔΔCt^) method for relative quantitation of gene expression.

### Overexpression of miR-141 and miR-720

2 × 10^5^ PANC-1 cells were transfected with mirVana miRNA Mimics (micro-RNA-141 and 720) or miRVana mimic Neg#1 (Ambion-Thermo Fisher Scientific) at a final concentration of 30pmol using Lipofectamine 3000 (Thermo Fisher Scientific) following the manufacturer’s protocol.

### Western blot analysis

Western blot analysis was performed by standard methods as described previously [56] using the following antibodies: rabbit anti human ZEB-1 antibody was used at a dilution of 1:500 (Abcam, ab64098); a mouse anti-human TWIST-1antibody (Abcam- ab50887) was used at a dilution of 1:250; a rabbit anti-human E-cadherin antibody (Abcam-ab53033) was used at a dilution of 1:500, a rabbit anti-human MAP4K4 antibody (Abcam-ab15583) was used at a dilution of 1:1000, a mouse anti-human JNK antibody (Santa cruze-sc7345) was used at a dilution of 1:500 and a mouse anti-human p-JNK antibody (Santa cruze-sc6254) was used at a dilution of 1:500 for overnight incubation.

### Cell proliferation assay

Cell proliferation was measured using the XTT cell proliferation kit (Biological Industries, Israel) according to the manufacturer’s instructions. Briefly, cells (5,000/well) were collected and seeded in 96-well plates and incubated at 37°C after transfection. Absorbance was measured at a wavelength of 450-500 nm, and relative cell growth was expressed as fold change relative to control cells.

### Cell migration/invasion assay

Transwell migration and invasion assays were conducted as described elsewhere [56]. Briefly, cell migration detection was evaluated using Transwell migration chambers (8 mm pore size; BD). Cell invasion detection was evaluated using Transwell migration chambers pre-coated with a layer of Matrigel (8 mm pore size; BD).

### Cell apoptosis analysis

For cell apoptosis analysis, cells were treated with increasing doses of gemcitabine (0.5-10 μM) [56] and stained with the Annexin V-FITC and PI Kit (MEBCYTO Apoptosis Kit, MBL) at 72-hours post transfection, according to the manufacturer’s instructions.

### Statistical analysis

Data were analyzed using the GraphPad software and expressed as mean ± SD. Differences between groups were assessed using Student’s *t*-test for significance. The correlation between N1 status and tumor miRNAs was analyzed using the log-rank test. Univariate Cox proportional hazards analysis was performed with positive nodal status as the dependent variable in order to construct a model for the prediction of nodal involvement. We assigned each patient a risk score according to a linear combination of the expression level of the miRNA, weighted by the regression coefficient from the training samples. All statistical tests were two-sided, and a *P* value ≤0.05 was considered a statistically significant difference.

## Abbreviations

PDAC: Pancreatic Ductal Adenocarcinoma
LN: Lymph Node
N0: Lymph node negative
N1: Lymph node positive
MiRNA: MicroRNA
EMT: epithelial-to-mesenchymal transition
FFPE: formalin-fixed paraffin-embedded
c-miRNAs: circulating microRNAs
